# Draft Genome Sequence of *Catellicoccus marimammalium*, a Novel Species Commonly Found in Gull Feces

**DOI:** 10.1128/genomeA.00019-12

**Published:** 2013-02-07

**Authors:** Michael R. Weigand, Hodon Ryu, Laura Bozcek, Konstantinos T. Konstantinidis, Jorge W. Santo Domingo

**Affiliations:** a School of Civil and Environmental Engineering; b School of Biology, Center for Bioinformatics and Computational Genomics, Georgia Institute of Technology, Atlanta, Georgia, USA; c National Risk Management Research Laboratory, U.S. Environmental Protection Agency, Cincinnati, Ohio, USA

## Abstract

*Catellicoccus marimammalium* is a relatively uncharacterized Gram-positive facultative anaerobe with potential utility as an indicator of waterfowl fecal contamination. Here, we report an annotated draft genome sequence that suggests that this organism may be a symbiotic gut microbe.

## GENOME ANNOUNCEMENT

*Catellicoccus marimammalium* was first isolated from the carcass of a porpoise that had succumbed to severe enteritis and peritonitis along the coast of Scotland (1). A low-G+C Gram-positive catalase-negative facultative anaerobe, *C. marimammalium* is closely related to, but biochemically distinct from, other genera within the order *Lactobacillales* (1). Recently, 16S rRNA gene-based surveys and species-specific PCR assays have shown high host distribution and an abundance of species closely related to *C. marimammalium* in different waterfowl (2–4). Migratory waterfowl facilitate the spread of microbial pathogens and are a notable source of fecal contamination in recreational waters. Thus, *C. marimammalium-*based methods are relevant to public health and to the environmental monitoring of waterfowl fecal contamination (3–5). However, the study of *C. marimammalium* remains limited, as isolating this species from environmental waters has not been possible using media that support the growth of other facultative anaerobic lactobacilli.

To shed light on the metabolic potential of *C. marimammalium* (strain CCUG 49459 T; Culture Collection, University of Göteborg, Sweden), genome sequence data were generated by a combination of Illumina HiSeq 2000 and Roche 454 platforms. All sequence reads were filtered and trimmed at both the 5′ and 3′ ends based on a threshold of *Q* = 20. Passed Illumina reads were assembled first in parallel runs of Velvet v.1.0.13 (6) with a range of k-mer coverage values (7). The resulting contigs were pooled and then assembled as pseudoreads in conjunction with passed 454 reads using the Genome Sequencer (GS) *de novo* Assembler software v.2.0.01.14 (Roche). This hybrid approach yielded 31 large contigs (≥500 bp each) with an N_50_ size of 153,794 bp, an average length of 41,619 bp, and a maximum length of 308,478 bp. The resulting genome sequence is 1,290,194 bases with 235-fold coverage and has an average G+C content of 42.0%. Annotation by the Rapid Annotations using Subsystems Technology (RAST) server (8), tRNAscan-SE (9), and RNAmmer (10) revealed 1,200 protein-coding regions, 48 tRNA genes, and a single rRNA operon.

Core-genome phylogeny confirmed the placement of *C. marimammalium* within the order *Lactobacillales*, where it forms a unique clade, which is consistent with previous 16S gene phylogenetic analyses (1). Preliminary reconstruction using Pathway Tools v.16 (11) revealed an overall reduced metabolic network with a particularly limited capacity for *de novo* amino acid biosynthesis compared to other closely related organisms. Rather, the genome of *C. marimammalium* encodes functions commonly found in symbiotic gut bacteria, such as bile acid hydrolysis and specialized nutrient transport. Taken together, the reduced genome and metabolic network suggest a symbiotic lifestyle that likely underlies the difficulty of growing *C. marimammalium* in synthetic media. The frequent detection of *C. marimammalium-*like sequences in different waterfowl feces (2–5) suggests that the physiology of this bacterial group is better adapted to waterfowl gut environment than to the gut of other potential hosts. The genome sequence reported here will aid in future studies of *C. marimammalium* to elucidate the nature of its gut symbiosis and to develop additional molecular tools for tracking fecal contamination in recreational waters.

### Nucleotide sequence accession numbers.

This Whole Genome Shotgun project has been deposited at DDBJ/EMBL/GenBank under the accession no. AMYT00000000. The version described in this article is the first version, AMYT01000000.
